# Predicted luteal phase length has no influence on success of vitrified-warmed blastocyst transfer in natural cycle

**DOI:** 10.1186/s13048-018-0436-6

**Published:** 2018-08-02

**Authors:** M. Reljič, J. Knez

**Affiliations:** 1 Department for Reproductive Medicine and Gynaecological Endocrinology, Clinic for Gynecology and Perinatology, University Medical Centre Maribor, Ljubljanska 5, 2000 Maribor, Slovenia; 2 Department for Reproductive Medicine and Gynaecological Endocrinology, Clinic for Gynecology and Perinatology, University Medical Centre Maribor, Ljubljanska 5, 2000 Maribor, Slovenia

**Keywords:** Predicted luteal phase length, Frozen embryo transfer, Blastocyst transfer, Natural cycle

## Abstract

**Background:**

This study evaluated the influence of menstrual cycle length, menstrual cycle variability and predicted luteal phase length on the success of vitrified-warmed blastocyst transfer in natural menstrual cycle using progesterone for luteal phase supplementation.

**Methods:**

Consecutive women undergoing vitrified-warmed blastocyst transfer in natural menstrual cycle between January 2013 and December 2015 were included in this retrospective study. Patients’ characteristics, clinical data and data about menstrual cycle length in the last year were collected from our database. Predicted luteal phase length (LPL) was defined as the period starting at ovulation (one day after positive urinary LH test) and ending on the last day before predicted menses, based on women’s usual, minimal and maximal menstrual cycle length data. Logistic regression was used to identify the predictors significantly associated with live-birth.

**Results:**

A total of 1195 FETs (frozen-thawed embryo transfers) resulted in 457 (38.24%) clinical pregnancies, 82 (17.94%), miscarriages and 371 live births (31.04%). There were no statistically significant differences in menstrual cycle length, menstrual cycle variability, day of LH surge, day of FET and predicted LPL between FET cycles resulting in live birth and those not resulting in live birth. In the multivariate logistic regression model, only women’s age (OR 0.93, 95% CI: 0.90–0.96), transfer of morphologically optimal blastocysts (OR 2.17, 95% CI: 1.59–2.94) and endometrium thickness (OR 1.10, 95% CI: 1.03–1.17) were important independent prognostic factors for live birth.

**Conclusion:**

Menstrual cycle length, menstrual cycle variability and predicted LPL do not seem to be an important factor influencing live birth after FET in natural cycles with progesterone supplementation. Results of our study suggest that FET should not be cancelled if LH surge is detected before or after the predicted period in natural cycle with progesterone supplementation.

## Background

The transfer of frozen-thawed embryos (FET) has become an integral part of successful in vitro fertilization programs. With advances in cryopreservation techniques, this approach offers several important benefits to the patients, which include improved safety of the treatment and higher cumulative success rates. When transferring embryos, a receptive endometrium is a prerequisite for successful implantation and several protocols for endometrium preparation can be used in patients undergoing FET. One of the commonly used approaches is natural cycle (NC) monitoring, where detection of ovulation is the reference for timing of embryo thawing and transfer [1]. The main advantages of NC over other protocols are avoidance of using multiple medications and low cost, although the timing of ovulation increases scheduling difficulties and cancelation rates [2]. Success rate of FET in NC depends on the appropriate patient selection. This is partly because cycle monitoring in women with irregular menstrual cycles is less feasible and more likely to result in canceling the embryo transfer. But even in women with regular and ovulatory cycles, the spontaneous conception rates are affected by menstrual cycle characteristics, such as menstrual cycle length, variability and luteal phase length [3–5]. It has been shown that even an isolated episode of short luteal phase may have an influence on reduced immediate fecundity in natural conception [3]. There is currently no data available showing a possible role of luteal phase length on the success of FET. The aim of our study was to determine whether predicted luteal phase length has an influence on the live birth rate in women undergoing FET in NC.

## Methods

All women undergoing vitrified-warmed blastocyst transfer performed in natural cycle between 2013 and 2015 at the Department for Reproductive medicine and Gynecological Endocrinology, University Medical Centre Maribor were included in this retrospective study. According to our clinical practice all women undergoing FET are scheduled for a baseline pelvic ultrasound examination in the follicular phase of the menstrual cycle. Women with irregular menstrual cycles (< 24 or > 35 days), uterine pathology and hydrosalpinges visible on ultrasound were excluded from the analysis.

After observing the selection of the leading follicle and thickening of the endometrium on ultrasound, urinary LH tests were used twice daily to monitor the LH surge onset. FET was scheduled 5 or 6 days after positive morning and evening LH test. If the LH surge was not detected or if the results were inconclusive, the embryo transfer was canceled.

All expanded blastocysts were vitrified on day 5 or day 6 by using the combination of dimethyl sulfoxide (DMSO) and ethylene glycol cryoprotectants. Before vitrification blastocysts were graded according to our established grading system [6, 7]. In brief, the blastocyst was considered optimal if it was fully expanded and the blastocoel completely filled the embryo. Blastocysts were first exposed to equilibration media (7.5% DMSO/ethylene glycol) for 10 min and later to vitrification solution (15% DMSO/ethylene glycol) for 1 min (Irvine Scientific, Santa Ana, CA). Before vitrification, blastocysts were placed in closed straws (High security vitrification kit, Cryo Bio System, Paris, France). For thawing, the embryo carrier was expelled from the straw and quickly plunged into the thawing medium (1 M sucrose) for 1 min. Blastocysts were then transferred in dilution solution (0.5 M sucrose) 3 times for 3 min. They were cultured in recovery medium (Blast Assist System, Origio, Denmark) for at least 4 h before they were transferred into the uterus. Only blastocysts that had at least 50% intact blastomeres after thawing and started to re-expand were assessed suitable for transfer [7].

One or two vitrified-warmed blastocysts were transferred using clinical touch technique and Labotect catheters (Labotect GmbH, Labor-Technik-Göttingen, Germany). The number of embryos transferred in each case depended on the quality of available embryos, the number of previous treatments, the number of embryos frozen in the same straw and according to the patient - doctor agreement.

Immediately prior to embryo transfer, pelvic ultrasound examination was performed to measure the endometrial thickness and evaluate the endometrial pattern. The endometrium thickness was measured in sagittal plane from one basal endometrial interface across the endometrial canal to the other basal surface. Secretory endometrial pattern was defined as an isoechoic and homogeneous hyperechoic endometrium with a non-prominent or absent central echogenic line.

According to our routine clinical practice, progesterone supplementation with 400 mg of micronized vaginal progesterone per day was started after FET. Serum β-hCG level measurement was scheduled 14 days after FET and ultrasound was performed 2 weeks later if the β-hCG levels were positive. Clinical pregnancy was defined as the presence of a gestational sac with a fetal heartbeat. Spontaneous miscarriage was defined as pregnancy loss after clinical confirmation of pregnancy. Live birth was recorded where one or more babies were born. The data on pregnancy outcome was collected by using a questionnaire.

Patients’ characteristics clinical data and cycle outcome was collected from our database. The data about menstrual cycle length (usual, shortest, and longest) in the last year was collected as part of the routine infertility assessment. Predicted luteal phase length (LPL) was defined as the period starting at ovulation (one day after positive urinary LH test) and ending on the last day before predicted menses, based on women’s usual, minimal and maximal menstrual cycle length data. A normal LPL was defined as 12–15 days, short as less than 12 days and long more than 15 days.

Patients’ and cycles’ characteristics were compared between the FET cycles resulting in live birth and FET cycles not resulting in live birth. Statistical analysis was performed with Statistica 8.0 data software system analysis (Stat Soft Inc., Tulsa, OK, USA). Mean and standard deviation for each continuous variable were calculated and Student’s t test was used to compare these variables between both groups. Chi-square test was used in the evaluation of the categorical data. We have constructed univariate logistic regression models to test the predictive values of different patients’ characteristics on the possibility of live birth. Variables proven statistically important by univariate logistic analysis were tested with multiple logistic regression model. Odds ratios and their 95% confidence intervals (CIs) were calculated. Live birth rates between cycles with predicted short, normal and long LPL were compared using Pearson chi-square test. *P* value < 0.05 was considered statistically significant.

The study was approved by our institutional review board.

## Results

A total of 1195 FETs resulted in 457 (38.24%) clinical pregnancies, 82 (17.94%), miscarriages and 371 live births (31.04%). Women in cycles resulting in live birth were statistically significantly younger (33.20 ± 3.97 vs 34.46 ± 4.14 years, *p* < 0.001), had less previous unsuccessful IVF/ICSI attempts (1.68 ± 1.35 vs 1.87 ± 1.43, *p* = 0.03), thicker endometrium on the day of FET (10.38 ± 2.34 vs 9.95 ± 2.17 mm, *p* = 0.004), higher proportion of transferred blastocysts vitrified on day 5 (71.70% vs 64.56%, *p* = 0.015) and higher proportion of FET of optimal blastocysts (38.27% vs 20.14% *p* < 0.001) compared to women in cycles not resulting in live birth. There were no statistically significant differences between these two groups in the rate of secretory endometrial pattern, number of blastocysts transferred, cause of infertility, proportion of cycles with births after fresh embryo-transfer and in proportion of cycles with FET after freeze-all cycle (Table 1). There were also no statistically significant differences in usual, shortest and longest menstrual cycle length, menstrual cycle variability (maximum- minimum MC length), day of LH surge, day of FET and predicted LPL between FET cycles resulting in live birth and those not resulting in live birth (Table 2).

**Table 1:** Clinical characteristics of FET cycles resulting in live birth and those not resulting in live birth

	FET resulting in live birth *N* = 371	FET NOT resulting in live birth *N* = 824	*p*-value
Age (mean ± SD)	33.20 ± 3.97	34.46 ± 4.14	< 0.001
Unexplained infertility (%, N)	17,52 (65)	15,29 (126)	NS
Tubal factor infertility (%, N)	22,37 (83)	26,46 (218)	NS
Male factor infertility (%, N)	42.31 (157)	42,84 (353)	NS
No. of previous IVF cycles (X ± SD)	1.68 ± 1.35	1.87 ± 1.43	0.03
Freeze all in previous cycle (%, N)	12.93 (48)	10.44 (86)	NS
Prior birth after fresh ET (%, N)	18.06 (67)	16.99 (140)	NS
No. of blastocysts transferred (X ± SD)	1.26 ± 0.44	1.23 ± 0.42	NS
ET of optimal blastocysts (%, N)	38.27 (142)	20.14 (166)	< 0.001
ET of blastocysts frozen on day 5 (%, N)	71.70 (266)	64.56 (532)	0.015
Endometrial thickness (mm, X ± SD)	10.38 ± 2.34	9.95 ± 2.17	0.004
Secretory endometrium pattern (%)	58.68	60.38	NS

*ET* embryotransfer

**Table 2:** Menstrual cycle characteristics in FET cycles resulting in live birth and those not resulting in live birth

	FET resulting in live birth N = 371	FET NOT resulting in live birth N = 824	p-value
Usual MC length (days)	28.17 ± 1.86	28.10 ± 1.67	NS
Min. MC length (days)	26.63 ± 2.42	26.74 ± 2.23	NS
Max. MC length (days)	30.20 ± 2.74	30.07 ± 2.70	NS
Max.- min. MC length (days)	3.68 ± 3.61	3.34 ± 2.68	NS
Day of cycle with LH surge	13.40 ± 2.16	13.32 ± 2.14	NS
Day of cycle with FET	19.26 ± 2.09	19.18 ± 2.09	NS
LPL predicted on usual MC length (days)	14.02 ± 1.92	13.91 ± 1.96	NS
LPL predicted on min. MC length (days)	12.41 ± 2.50	12.24 ± 2.71	NS
LPL predicted on max. MC length (days)	15.81 ± 2.53	15.73 ± 2.71	NS

*MC* menstrual cycle, *LPL* luteal phase length, *min* minimal, *max* maximal

In the multivariate logistic regression model, only women’s age (OR 0.93, 95% CI: 0.90–0.96), transfer of morphologically optimal blastocysts (OR 2.17, 95% CI: 1.59–2.94) and endometrium thickness (OR 1.10, 95% CI: 1.03–1.17) were important independent prognostic factors for live birth.

In 185 (15.48%) FET cycles, the predicted LPL was short (≤ 11 days), in 952 (79.67%) normal (12–15 days), and in 58 (4.85%) long (> 15 days) if the calculation was based on usual menstrual cycle length. If cycle variability was taken into account then the proportion of predicted short, normal and long luteal phase length was different. However, there were no significant differences in live birth rate between these three groups, irrespective of whether the length of the luteal phase was predicted on the usual, minimum or maximum length (Table 3).

**Table 3:** Predicted luteal phase length in minimal, usual and maximal menstrual cycle length according to live birth rate

	Predicted short LPL (≤11 days)	Predicted normal LPL (12–15 days)	Predicted long LPL (> 15 days)	*p*- value
LPL predicted on min. MC length (%, N)	32.05 (383)	59.50 (711)	8.45 (101)	
Live birth rate	33.43 (128)	29.67 (211)	31.68 (32)	NS
LPL predicted on usual MC length (%, N)	15.48 (185)	70.79 (846)	13.72 (164)	
Live birth rate	34.59 (64)	30.39 (257)	30.49 (50)	NS
LPL predicted on max. MC length (%, N)	2.59 (31)	47.95 (573)	49.46 (591)	
Live birth rate	32.25 (10)	30.54 (175)	31.47 (186)	NS

*MC* menstrual cycle, *LPL* luteal phase length, *min* minimal, *max* maximal

## Discussion

Our data has shown that the predicted luteal phase length is not predictive of live birth after FET. The only important independent prognostic factors for live birth were women’s age at the time of FET, transfer of morphologically optimal blastocysts and endometrial thickness, a finding that was also reported in previous studies [8, 9].

There are currently no other studies evaluating the influence of menstrual cycle characteristics on the outcome of FET in NC, but some observations that support this possibility come from studies on natural fertility. Based on these studies, menstrual cycle pattern, menstrual cycle length and variability are indicators of endocrine function and fertility potential of women. Usually menstrual cycle shortens with chronologic age and then there is an increase in variability at perimenopause [10]. Several authors have studied the association between menstrual cycle characteristics and spontaneous conception rate and discovered that shorter cycles are associated with reduced fecundability in women with regular cycles [4, 5, 10]. However, it was shown that adverse effect of short menstrual cycle length on fecundability was more pronounced in women with cycle length less than 25 days [10] and these women were not included in the present study.

Normal luteal phase length (LPL) is relatively fixed at 12–14 days [11]. Short LPL is often considered to be a clinical sign of luteal phase deficiency (LPD), which is an entity commonly associated with infertility [11]. LPD can be caused by impaired corpus luteum function, resulting in the lack of adequate progesterone secretion or by inadequate response of endometrium to progesterone. Progesterone secretion in the luteal phase of menstrual cycle is crucial for secretory transformation of the endometrium leading to receptivity [12]. Therefore, women with short luteal phase may have impaired receptivity of the endometrium to implantation or lower capability to maintain a pregnancy [13]. Short luteal phase is not uncommon and was observed in 8.9–18.0% among normal cycling woman. In the present study, short luteal phase was predicted in 15.5% of cycles if calculation was based on woman’s usual menstrual cycle length data. But if we take into account the menstrual cycle variability, the rate of cycles with predicted short luteal phase was between 2.6–32.0%. But regardless of the approach to calculation of the predicted LPL, there were no statistically significant differences between cycles with predicted short, normal and long luteal phases. One of the possible explanations could be that in the present study, luteal phase support was used. So even if LPD existed, it could be overcome by using micronized progesterone after embryo transfer. The benefit of luteal phase support in natural cycles has previously been demonstrated by Bjuresten et al. [14]. They concluded that women undergoing FET are often subfertile, and they may have suboptimal endometria during their natural cycles [14]. On the other hand, there is controversy in this field, since other authors did not confirm these findings and Groenewoud et al. in their meta-analysis concluded that currently there is too little evidence supporting a positive effect of luteal phase support in patients undergoing NC-FET [1, 15].

Limitations of the present study are its retrospective nature. The calculated predicted LPL is an entity that indirectly estimates the luteal phase length of the current cycle and this is a limitation of our study. Despite using multivariate regression models in the methodological approach to control for confounders, the presence of potential bias cannot be excluded. Self-reported data on menstrual cycle length and urinary LH tests could be unreliable. Luteal phase has been affected by progesterone supplementation after FET.

## Conclusion

Menstrual cycle length, menstrual cycle variability and predicted LPL do not seem to play an important role for live birth rate after FET in natural cycles with progesterone supplementation. Results of our study also suggest that FET should not be cancelled if LH surge is detected before or after predicted period in natural cycle with progesterone supplementation. There is a need for further basic studies investigating the influence of luteal phase length on embryo implantation.
